# Nonadaptive molecular evolution of seminal fluid proteins in *Drosophila*

**DOI:** 10.1111/evo.14297

**Published:** 2021-07-09

**Authors:** Bahar Patlar, Vivek Jayaswal, José M. Ranz, Alberto Civetta

**Affiliations:** ^1^ Department of Biology University of Winnipeg Winnipeg MB R3B 2E9 Canada; ^2^ School of Mathematics and Statistics The University of Sydney Sydney NSW 2006 Australia; ^3^ Department of Ecology and Evolutionary Biology University of California Irvine Irvine California 92697

**Keywords:** Functional attributes, nonadaptive evolution, postcopulatory sexual selection, relaxed selection, selective constraints, seminal fluid proteins

## Abstract

Seminal fluid proteins (SFPs) are a group of reproductive proteins that are among the most evolutionarily divergent known. As SFPs can impact male and female fitness, these proteins have been proposed to evolve under postcopulatory sexual selection (PCSS). However, the fast change of the SFPs can also result from nonadaptive evolution, and the extent to which selective constraints prevent SFPs rapid evolution remains unknown. Using intra‐ and interspecific sequence information, along with genomics and functional data, we examine the molecular evolution of approximately 300 SFPs in *Drosophila*. We found that 50–57% of the SFP genes, depending on the population examined, are evolving under relaxed selection. Only 7–12% showed evidence of positive selection, with no evidence supporting other forms of PCSS, and 35–37% of the SFP genes were selectively constrained. Further, despite associations of positive selection with gene location on the X chromosome and protease activity, the analysis of additional genomic and functional features revealed their lack of influence on SFPs evolving under positive selection. Our results highlight a lack of sufficient evidence to claim that most SFPs are driven to evolve rapidly by PCSS while identifying genomic and functional attributes that influence different modes of SFPs evolution.

Reproductive genes typically evolve more rapidly than nonreproductive genes, and seminal fluid proteins (SFPs) encoding genes are considered to be among those evolving the fastest, with loss of detectable orthologs between related species (Swanson et al. 2001; Swanson and Vacquier 2002; Haerty et al. 2007; Dean et al. 2009; Wilburn and Swanson 2016; Rowe et al. 2020). Because of the diversity of SFP functions related to both male and female reproductive fitness, the rapid evolution of SFPs has been primarily attributed to postcopulatory sexual selection (PCSS) driving divergence between species. PCSS can result from directional forms of selection, like positive selection conferring some SFP variants a net advantage in ejaculate function, or forms of sexual conflict leading to an escalating coevolutionary chase between the sexes (Sirot et al. 2015; Sayadi et al. 2019; Rowe et al. 2020; Wigby et al. 2020). For example, phylogenetic approaches comparing species with different mating strategies (e.g., monandrous vs. polyandrous) have demonstrated a positive relationship between the intensity of sperm competition and positive selection on SFP genes (Kingan et al. 2003; Dorus et al. 2004; Walters and Harrison 2011; reviewed by Wong 2011). However, these studies often do not consider that mating system can be seasonally or resource availability dependent, and that more than one mating system are common within a single species (Dixson 1999; Maher and Burger 2011).

Using molecular population genetics approaches, studies focused on a single or few SFP genes found evidence of a rapid adaptive evolution by positive selection (Aguadé et al. 1992; Tsaur and Wu 1997; Aguadé 1999; Begun et al. 2000; Holloway and Begun 2004; Haerty et al. 2007). Nevertheless, the generality of this finding remains questionable. Molecular evolution studies of large numbers of SFPs have supported an enrichment for positive selection compared to other genes, but the studies have found only 11–15% of all SFPs evolving under positive selection (Swanson et al. 2001; Clark and Swanson 2005; Rowe et al. 2020). This low percentage of positively selected genes is compatible with a variety of selective constraints acting on male reproductive proteins and with the intensity of such constraints being dependent on each protein function (Dean et al. 2009; Carnahan‐Craig and Jensen‐Seaman 2014; Sirot 2019).

Recently, the notion that the high rate of evolution of reproductive genes is primarily the result of PCSS has been challenged, pointing to relaxed selection as a more suitable alternative explanation (Dapper and Wade 2016, 2020). First, reproductive genes show sex‐biased expression, which effectively means that the action of selection is limited, or happens primarily, in only one sex (Ranz et al. 2003; Ellegren and Parsch 2007). Second, mechanisms through which sexual selection operates, such as sperm competition, would not be associated—as often assumed—with strong selection coefficients as their outcome depends on the genotype of both males and females (Clark 2002; Dapper and Wade 2016, 2020). Third, the underlying role of reproductive genes in adaptation is often evaluated by determining whether there is an elevated ratio of nonsynonymous to synonymous substitutions (i.e., *d*_N_/*d*_S_, also known as *K*_a_/*K*_s_ or *ω*) (Swanson et al. 2001; Findlay et al. 2008), without incorporating information on populations polymorphisms into the analysis. The lack of polymorphism data complicates interpretations about the specific role of selection (Dapper and Wade 2020).

Prior studies have mainly focused on rates of evolution between species of variable numbers of SFPs. In addition, only a limited number of studies have jointly analyzed population polymorphism and divergence to tease apart the role of different selective pressures on the evolution of a handful of SFPs. The limited number of genes assayed and a reliance on interspecies phylogenetic comparisons to infer selection, along with a tendency to frame selection (e.g., positive selection) as the null hypothesis, instead of neutrality, have in some cases complicated results interpretation. *Drosophila* offers the opportunity to test hundreds of SFPs using statistical tests that use population polymorphism data along with divergence relative to a closely related species, and model synonymous changes as selectively neutral mutations formally allowing to infer deviations due to PCSS (Kreitman 2000; Nielsen 2005). Here, we scrutinize whether SFPs are rapidly evolving and whether PCSS, relaxed selection, or selective constraints have dominated their evolution. Additionally, by using data from an ancestral African (Zambia) and a derived North American (Raleigh) population of *Drosophila melanogaster*, we evaluate their commonalities and differences between populations. Finally, the richness of information on *Drosophila* SFPs in terms of genomics and functional features provides a unique opportunity to uncover relevant underlying factors contributing to our evolutionary findings.

## Materials and Methods

### SEMINAL FLUID PROTEINS

Given their potential role in PCSS, we used a list of 291 SFPs transferred during mating (Wigby et al. 2020). Additionally, we identified the 50 most highly expressed accessory gland genes according to FlyAtlas2 (http://flyatlas.gla.ac.uk/FlyAtlas2/index.html). Out of these 50 genes, 24 were already in the list of transferred SFPs (Wigby et al. 2020). The remaining 26 were catalogued as nontransferred SFPs based on data from previous proteomics studies (Findlay et al. 2008; Sepil et al. 2019; Wigby et al. 2020), and we used these nontransferred SFPs as a comparative group against transferred SFPs. For each gene, we retrieved functional data for three categories we considered likely to be enhanced for PCSS: immune function genes (due to host‐pathogen interaction), proteases (protein‐protein interactions and SFP processing related to function), and SFPs triggering postmating effects (e.g., male × male × female interactions). Information about immune function and protease activity was gathered from Wigby et al. (2020) and Biological Process GO from Gene Ontology Consortium (http://geneontology.org/). For postmating effect, we used information based on SFPs known role in sperm competition (Civetta and Ranz 2019) and supplemented the list with SFPs of known reproductive function that impact postmating phenotypes and those with roles in mating plug formation (Wigby et al. 2020). Transcript expression levels were retrieved from FlyAtlas2 (Leader et al. 2018), and we used expression values to calculate gene's tissue specificity index (Tau Index, *τ*) (Yanai et al. 2005). We categorized genes as tissue specific if *τ* was ≥0.9 in either the accessory glands (AG), the testes (T), or any other tissue. Lastly, reliable age estimates on the origin of each gene within the *Drosophila* phylogeny were retrieved as reported (Xia et al. 2020). Using this information, we categorized genes into five age classes, with older genes being those genes present in species of the genus *Drosophila*, followed by genes originated in the ancestor to the subgenus *Sophophora*, the *melanogaster* group, the *melanogaster* subgroup, and unique to *D. melanogaster*, or shared with species of the *simulans* complex.

### GENOMIC DIVERSITY DATA

We retrieved population genomics and interspecies divergence data from the iMKT web‐based service page (https://imkt.uab.cat/) (Murga‐Moreno et al. 2019). The population genomics data come from 197 lines, mostly isofemale, derived from a Zambia (ZI) African population, and 205 inbred lines from the North American population of Raleigh (RAL), NC (MacKay et al. 2012; Huang et al. 2014; Lack et al. 2016). We extracted estimates on per gene Derived Allele Frequencies (DAF) and the number of synonymous and nonsynonymous polymorphic and divergent (relative to *D. simulans*) sites and changes. All data were extracted from 13,753 protein encoding genes using the iMKT R package (Murga‐Moreno et al. 2019).

### MOLECULAR EVOLUTION ANALYSES

First, we calculated SFPs rate of evolution (absolute divergence, *D_xy_*) (Nei and Li 1979) as the proportion of nucleotide substitutions between species. Second, we used the ratio of nonsynonymous substitutions per nonsynonymous site to synonymous substitutions per synonymous site (*K*_a_/*K*_s_) to identify SFPs under the action or not of selective constraints. Both *D_xy_* and *K*_a_/*K*_s_ estimates were compared between SFPs and the rest of the genome using a nonparametric Wilcoxon Rank Sum test (Wilcoxon 1945). Third, we used the extended McDonald‐Kreitman Test (eMKT) method (MacKay et al. 2012), which separates counts of segregating sites in the nonsynonymous class into neutral and weakly deleterious variants to estimate the ratio of substitutions to polymorphism between nonsynonymous and synonymous sites (*α*) (Smith and Eyre‐Walker 2002). The eMKT analysis was used to identify SFPs under positive selection, or with a significant excess of slightly deleterious nonsynonymous polymorphisms, after *P*‐values were False Discovery Rate (FDR) corrected (Benjamini and Hochberg 1995). Moreover, we used this method to obtain estimates of the ratio of both adaptive (*ω*_a_) and nonadaptive (*ω*_na_) nonsynonymous substitutions to synonymous substitutions. Finally, the mean ratio of nonsynonymous to synonymous polymorphisms (*π*_a_/*π*_s_) and divergence (*K*_a_/*K*_s_) were calculated for SFP genes and the remainder of the genome, while correcting for covariance between the two estimates. We used bootstrapping to estimate 95% confidence intervals and used these estimates to evaluate patterns of polymorphism and divergence expected under different forms of selection (Dapper and Wade 2020).

### FUNCTIONAL AND GENOMIC ANALYSES

To test the degree of association between different genomic or functional features and the mode of SFP evolution, we applied two different statistical approaches. Categorical features related to biological processes and functions (i.e., transfer to the female reproductive tract; tissue of expression; immune function; postmating effect; proteases), or to genome properties (sex vs. autosomal location; and phylogenetic age), were tested for nonrandom associations with predictive modes of SFP evolution using Fisher exact tests (FETs). All these calculations and statistical analyses were done in R Statistical Software version 3.5.3 (R Development Core Team 2017).

For noncategorical variables, we selected features that can affect the outcome of selection tests (gene length, number of transcripts, codon bias, *τ*, recombination frequency), and a multiclass classifier was developed to predict their mode of evolution. Variables with statistically significant differences among SFPs categorized based on mode of evolution were first identified using one‐way analysis of variance tests. Second, stratified random sampling was performed to split the input data (comprising the predictor variables identified above and the output class) into training and test set in the ratio 2:1, generating 100 cross‐validation datasets. Third, the predictor variables were standardized to ensure that each variable had a mean value of 0 and standard deviation of 1. Lastly, a multinomial logistic regression model was developed using the training set and evaluated on the test set. Overall accuracy was chosen as the evaluation metric and was defined as the ratio *M*/*N*, where *N* denotes the number of observations in the test set and *M* denotes the number of observations whose class was predicted correctly.

Subsequently, we determined the importance of the predictor variables using likelihood ratio tests. First, the complete dataset (i.e., the dataset comprising all the predictor variables) was used to estimate the log‐likelihood of the model. Second, log‐likelihoods were obtained for simpler models with one of the predictor variables removed. Lastly, likelihood ratio tests were performed to test the null hypothesis that the difference between the log‐likelihood for the complete model and a simpler model was explained by the difference in the number of model parameters. We ranked the predictor variables based on the *P*‐values of likelihood ratio tests, such that the variable with the smallest value was considered to be the most important in predicting the mode of evolution.

The multivariate statistical analyses were performed in Python using in‐home scripts, which can be found in Dryad (https://datadryad.org) at https://doi.org/10.5061/dryad.rjdfn2zbg.

## Results

### DO SFP GENES EVOLVE RAPIDLY?

We grouped 317 SFP genes (Table S1) into fast and nonfast evolving by comparing their divergence (*D_xy_*) against the rest of the genome (Fig. S1). We found that the average divergence of SFP genes is markedly higher (ZI = 0.051 and RAL = 0.048) than the rest of the genome (ZI = 0.032 and RAL = 0.029) (Table 1). Moreover, 65–66% (ZI = 205/317 and RAL = 210/317) of all SFPs have higher *D_xy_* than the upper limit CI of the rest of the genome. Nevertheless, it is noticeable that a relatively large proportion of SFPs (32–33%) in both populations (ZI = 105, RAL = 100) evolve at rates below the genome average (Fig. S1).

**Table 1:** A comparison of *D. melanogaster* and *D. simulans* divergence between SFPs and the rest of the genome

Pop.	Genome			SFPs			Comparisons	
	*n*	Mean	95% CI	*n*	Mean	95% CI	*W*	*P*‐value
Zambia								
*D_xy_*	13,370	0.032	0.031–0.032	317	0.051	0.047–0.056	12.6 × 10^5^	<0.001
*K*_a_	13,370	0.022	0.021–0.022	317	0.042	0.037–0.047	12.3 × 10^5^	<0.001
*K*_s_	13,302	0.074	0.073–0.075	315	0.089	0.084–0.095	16.5 × 10^5^	<0.001
*K*_a_/*K*_s_	12,801	0.274	0.268–0.279	310	0.474	0.426–0.524	12.6 × 10^5^	<0.001
Raleigh								
*D_xy_*	13,370	0.029	0.028–0.029	317	0.048	0.048–0.059	13.3 × 10^5^	<0.001
*K*_a_	13,370	0.019	0.018–0.019	317	0.039	0.034–0.044	13.1 × 10^5^	<0.001
*K*_s_	13,302	0.071	0.070–0.071	315	0.089	0.082–0.096	16.6 × 10^5^	<0.001
*K*_a_/*K*_s_	12,801	0.251	0.245–0.256	283	0.443	0.396–0.490	10.3 × 10^5^	<0.001

Means and 95% confidence intervals (CI) from 10,000 bootstraps for *D_xy_*, *K*_a_, *K*_s_, and *K*_a_/*K*_s_ for genome and SFP genes are shown. Wilcoxon Rank Sum (*W*) tests were applied to assess for differences in divergence between SFP genes and the rest of the genome. *n* is the number of proteins examined.

### IS EVOLUTION OF SFP DRIVEN BY PCSS?

**Figure 1:**
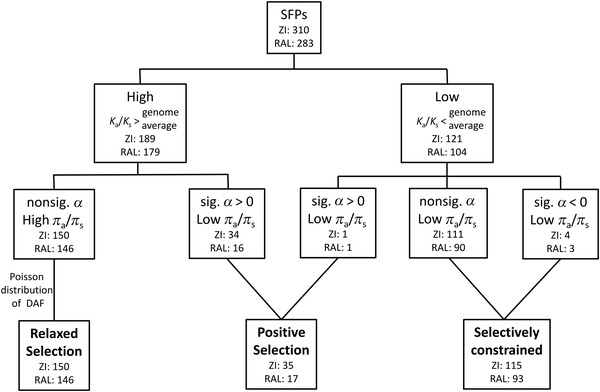
Mode of evolution of SFP genes. Genes were divided into two groups based on their *K*_a_/*K*_s_ ratios relative to the genome average. The eMKT, as well as comparisons of polymorphism (*π*_a_/*π*_s_) and divergence (*K*_a_/*K*_s_), and the frequency distribution of derived alleles (DAF) were used to group genes under three major groups (bold). Significance (sig.) indicates FDR corrected *P* < 0.05.

The *K*_a_/*K*_s_ ratio has been traditionally used as a proxy to detect selection (positive vs. negative or purifying). We calculated *K*_a_/*K*_s_ for all genes except for those with zero *K*_s_ (635 and 1923 genes in the genome and seven and 34 genes SFPs for ZI and RAL populations, respectively) (Table S1). We found that the average *K*_a_/*K*_s_ for SFP genes and the rest of the genome are about the same for both populations, but the SFPs average ratio was significantly higher than that for the genome (Table 1; Fig. S1). Approximately 61–63% of SFP genes (ZI: 189/310 and RAL: 179/283) had higher *K*_a_/*K*_s_ than the rest of the genome. This pattern of increased nonsynonymous substitutions between species is compatible with relaxed or positive selection (Fig. 1).

The MK test allows to jointly evaluate polymorphism and divergence by considering synonymous changes as neutral and testing for departures driven by excesses in the proportion of either nonsynonymous divergence or polymorphism. A significant excess of amino acid divergence (*α* > 0) is indicative of adaptive diversification between species, whereas a significant excess of amino acid polymorphism (*α* < 0) is driven by the segregation of slightly deleterious nonsynonymous mutations (Fig. 1; Table S1). Although the eMKT could not be run for 10 (ZI) and 27 (RAL) genes due to lack of polymorphism (Table S1), we uncovered that only 7% (RAL: 17/256) and 12% (ZI: 35/300) SFPs show a significant increase in nonsynonymous substitutions relative to polymorphism (significant positive *α* values at 5% FDR). This result is consistent with a pattern expected under positive selection (Fig. 1).

**Figure 2:**
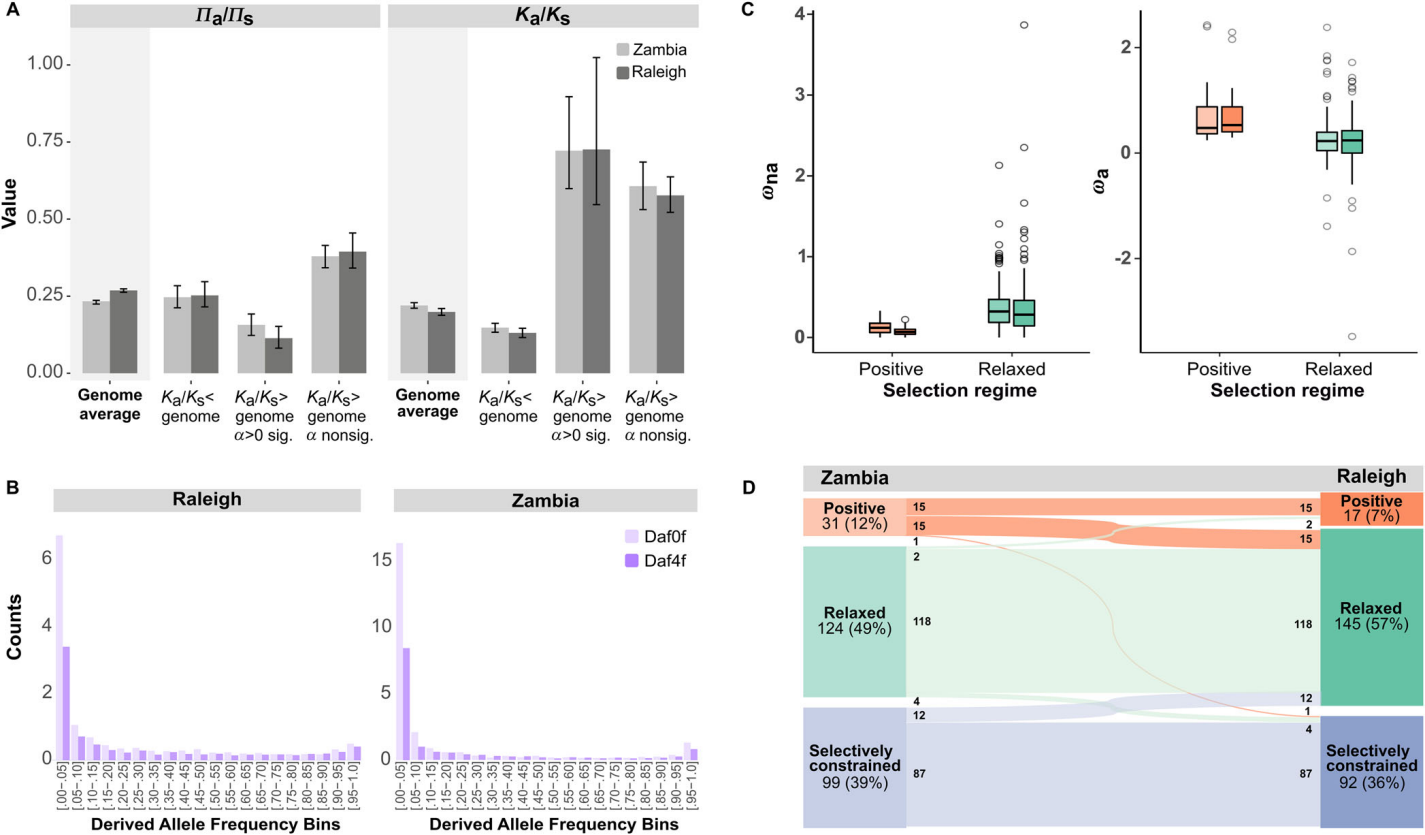
Summary statistics for polymorphism and divergence, and modes of evolution, compared between the ancestral (ZI) and derived (RAL) *D. melanogaster* populations. (A) Average polymorphism (*π*_a_/*π*_s_) and divergence (*K*_a_/*K*_s_) for the genome and SFPs after correcting for covariance between the two estimates. Error bars are 95% confidence intervals. (B) The derived allele frequency spectrum of mean counts of nonsynonymous (Daf0f) and synonymous (Daf4f) polymorphisms in SFP genes with *K*_a_/*K*_s_ higher than the genome average and nonsignificant *α*. (C) The ratio of nonadaptive (*ω*_na_) and adaptive (*ω*_a_) nonsynonymous to synonymous substitutions, respectively. Error bars represent standard error of the mean. Dark‐colored bar: Raleigh population, light‐colored bar: Zambia population. (D) SFP genes modes of evolution in ZI and RAL. Modes of evolution groups are positive selection (orange), relaxed selection (green), and selectively constrained (blue). Numbers are counts per group with colored lines showing movement of genes across group classifications.

Different patterns of polymorphism and divergence are expected under different selective regimes. Positive selection predicts high *K*_a_/*K*_s_ but low polymorphism (*π*_a_/*π*_s_), whereas relaxed selection and sexual conflict predict high *K*_a_/*K*_s_ and high *π*_a_/*π*_s_ (Fig. 1) (Kreitman 2000; Nielsen 2005; Dapper and Wade 2020). Using genome estimates as a control, we found that SFPs identified as evolving under positive selection show the expected pattern of low *π*_a_/*π*_s_ and high *K*_a_/*K*_s_ (Figs. 2A and S2), whereas others that were not identified as evolving under positive selection show high *K*_a_/*K*_s_ and high *π*_a_/*π*_s_ (Figs. 2A and S2). Thus, the evolution of SFPs with *K*_a_/*K*_s_ estimates higher than the genome average, which did not depart from the neutral expectation (Fig. 1; nonsignificant *α*), is consistent with patterns of polymorphism and divergence expected under relaxed selection or sexual conflict.

Sexual conflict involves negative synergism between sexes and predicts the maintenance of intermediate‐frequency polymorphisms, whereas relaxed selection should produce a distribution with a large number of low‐frequency alleles (Ewens 1972; Wagner 2007; Kasimatis et al. 2017; Dapper and Wade 2020). We observed that SFPs with high *K*_a_/*K*_s_ and high *π*_a_/*π*_s_ show a distribution of alleles frequencies in accordance with expectation for relaxed selection (Fig. 2B). We further compared genes under positive and relaxed selection for the ratio of adaptive and nonadaptive nonsynonymous to synonymous substitutions. We found that genes under relaxed selection have significantly more nonadaptive nonsynonymous (Wilcoxon Test; ZI = 827.5, *P* < 0.001, RAL = 332, *P* < 0.001) and fewer adaptive nonsynonymous to synonymous substitutions (Wilcoxon Test; ZI = 4159, *P* < 0.001, RAL = 1934, *P* < 0.001) than genes under positive selection (Fig. 2C). Overall, our results support that a large number of SFP genes (ZI = 150; RAL = 146) have evolved under relaxed selection.

A potentially important caveat is that the ability to reject the null hypothesis of neutrality can be weak for short coding sequences, making cases of relaxed selection indistinguishable from cases of weak positive selection. When we iteratively removed the shortest genes from the dataset, we found no differences between mode of evolution and gene coding sequence length after eliminating the smallest third of the genes (Table S2). Moreover, the sample with only two thirds of the genes had no differences in the proportion of selectively relaxed genes in the sample relative to the whole dataset (Table S2). Once the length effect was removed, the proportion of relaxed genes remained larger than the proportion of positively selected genes (Table S2). For example, for ZI the percentage of relaxed and positively selected genes changed from 50% and 12% in the entire dataset to 41% and 15% after removing the shortest coding sequences, respectively.

Finally, a considerable proportion of SFPs (35–37%) in both populations (ZI = 121/300, RAL = 104/256) have *K*_a_/*K*_s_ ratios below the genome average, suggestive of a group of genes facing evolutionary constraints in interspecies divergence (Fig. 1; Table S1). One gene in this group (*CG9364*; *Trehalase*) was detected as a positively selected gene in both populations (Fig. 1). *Trehalase* is a nontissue‐specific gene involved in glucose metabolism and its SFP is transferred to females during mating. In addition, four genes in ZI (*CG42326*, *CG9294*, *CG33784*, and *Spn28Da*) and three others in RAL (*CG11598*, *CG17271*, and *Spn42Dd*) showed a significant excess of nonsynonymous polymorphisms relative to nonsynonymous substitutions (*α* < 0, *P*_adj._ < 0.05) (Fig. 1). This is consistent with an excess of slightly deleterious segregating variants contributing to polymorphism but not divergence or with variants previously under purifying selection that become effectively neutral, thus increasing polymorphism relative to divergence.

### POPULATION COMMONALITIES AND DIFFERENCES

Using a combination of polymorphism and divergence data, we identified SFP genes evolving under different selective regimes and we have grouped them into three main categories: positive selection, relaxed selection, and selectively constrained genes (Fig. 1). Although most genes showed similar patterns of evolution regardless of the population considered, we did find differences in the proportion of the three modes of evolution between the two populations (McNemar's *χ* ^2^ = 14.941, d.f. = 3, *P* = 1.9 × 10^−3^). We found proportionally more genes being selectively constrained or under positive selection in the ancestral (ZI) population, and for a fraction of these genes selection became relaxed in the derived (RAL) population (Fig. 2D).

### ARE GENOMIC AND FUNCTIONAL FEATURES PREDICTIVE OF THE SFP MODE OF EVOLUTION?

We tested for associations between seven categorical features and mode of evolution (i.e., positive selection, relaxed selection, or selective constraint). For the two genomic features and two of the functional features, we found clear evidence of nonrandom association. Positively selected genes were overrepresented on the X chromosome and relaxed selection was significantly associated with SFP genes present on autosomes (Table 2; two‐tailed FET, *P*_adj._ < 0.05). In no case were such genes physically clustered, that is, they were adjacent. Relative to the gene age, we used the phylogenetic dating of 13,083 genes of *D. melanogaster* (http://gentree.ioz.ac.cn/download.php). We categorized 258 SFP genes within five age classes following reliably inferred phylogenetic origins of the gene complement of *D. melanogaster* within the evolution of the genus *Drosophila* (Xia et al. 2020). Compared to the representation of such age classes across the whole gene repertoire, we found a disproportionately high number of SFP encoding genes that belong to relatively recent age classes and a scarcity of ancient genes, that is, those that arose before the split between the two main subgenera in the genus *Drosophila* or age class *Drosophila* genus (Fig. S3; *χ* ^2^ = 520.63, *P =* 1 × 10^−5^, 10,000 simulations). The subsequent examination of how these age classes were associated with the three different modes of evolution revealed a nonrandom interplay between both variables (ZI: *χ* ^2^ = 35.088, *P =* 5 × 10^−4^; RAL: *χ* ^2^ = 28.762, *P =* 2 × 10^−3^; 2000 simulations each). Although there is no significant association between age classes and the genes evolving under positive selection, we found an overrepresentation of relatively young genes (age class *melanogaster* subgroup) among those genes evolving under relaxed selection and an overrepresentation of ancient genes (age class *Drosophila* genus) among those evolving under constrained selection (Table S3). Therefore, it seems that in contemporary populations of *D. melanogaster*, gene age is not associated with adaptive evolution, younger genes are more likely to evolve under relaxed evolution, and ancient genes are more constrained in their mode of evolution.

**Table 2:** Patterns of nonrandom association for six genomic or functional features and different gene categories based on their mode of molecular evolution

		Selection regime					
		Constrained		Positive		Relaxed	
Feature	*P*‐value^1^	Odds ratio	*P*‐adj.^2^	Odds ratio	*P*‐adj.^2^	Odds ratio	*P*‐adj.^2^
Zambia							
Transferred vs. nontransferred	0.076	2.358	0.169	3.000	0.487	0.327	0.074
Autosomes vs. X	**<0.001**	1.123	1.000	0.109**↓**	**<0.001**	5.661**↑**	**0.005**
Reproductive vs. nonreproductive	**<0.001**	0.251**↓**	**<0.001**	1.174	0.833	3.813**↑**	**<0.001**
Post‐mating vs. unknown	1.000	1.036	1.000	0.871	1.000	1.022	1.000
Immunity vs. unknown	0.178	1.581	0.413	2.152	0.331	0.400	0.331
Proteases vs. nonproteases	**0.009**	1.062	1.000	3.745**↑**	**0.017**	0.431	0.057
Raleigh							
Transferred vs. nontransferred	0.076	2.358	0.169	3.000	0.487	0.327	0.074
Autosomes vs. X	**<0.001**	0.779	0.626	0.096**↓**	**0.001**	4.126**↑**	**0.010**
Reproductive vs. nonreproductive	**<0.001**	0.288**↓**	**<0.001**	1.275	0.784	3.168**↑**	**<0.001**
Post‐mating vs. unknown	0.583	0.776	0.686	0.655	0.768	1.396	0.686
Immunity vs. unknown	0.230	1.319	0.776	2.690	0.776	0.545	0.776
Proteases vs. nonproteases	**0.017**	0.803	0.693	5.112**↑**	**0.020**	0.671	0.503

Only the 254 SFPs common to the populations of Zambia and Raleigh are considered.

^1^For each feature, genes are split into two categories and differential association with the three modes of evolution is tested using a 2 × 3 Fisher exact test (FET). When significant, the *P*‐value is bolded.

^2^Post hoc 2 × 2 FETs to test for significant excess (odds ratio > 1) or deficit (odds ratio < 1) between any selective regime and the other two. *P*‐values are FDR corrected. When significant, the *P*‐value is bolded, and an arrow identifies the excess or deficit for the first category listed. For the alternative category, the pattern is the opposite (e.g., autosomal SFPs are underrepresented, whereas X‐linked SFPs are enriched, in the positive selection group).

Among the categorical functional features, we found a significant effect for tissue of expression and proteolytic function (Table 2; two‐tailed FETs, *P*_adj._ < 0.05). Additionally, a disproportionally large number of positively selected genes encode for proteases (Table 2). Relaxed selection was significantly overrepresented among the SFP genes exhibiting male‐specific tissue expression (Table 2). When we further examined male‐specific tissue‐expressed genes, we found significant differences in how accessory gland‐specific genes and those that are either broadly expressed or specific in expression in nonreproductive tissues were represented across the three modes of evolution. The results show an excess of AG‐SFPs among genes under relaxed selection, whereas other tissue‐specific and nontissue‐specific SFPs are selectively constrained (Table S4).

Relative to the noncategorical genomic and functional features, four out of five analyzed showed significant differences among the three modes of evolution (Table S5). Subsequently, these relevant features were used to develop a multiclass prediction model, which resulted in a mean overall accuracy of 0.59 for Zambia and 0.67 for Raleigh (Fig. S4). The addition of the discarded features did not improve the results. For both populations, we found that the classifier was not able to predict accurately the gene class evolving under positive selection, which is to some extent expected due to the small number of observations in this class. Notably, the relative contribution of the different predictor variables in relation to the relaxed and selectively constrained modes of evolution was inconsistent between populations.

## Discussion

Our analysis of an extended list of SFP encoding genes showed that most of these genes evolve, on average, faster than the rest of the genome, in good agreement with prior reports that highlighted their fast interspecific divergence (Swanson et al. 2001; Dorus et al. 2006; Ramm et al. 2009; Walters and Harrison 2010; Ahmed‐Braimah et al. 2017; Rowe et al. 2020). The fast evolution of SFPs has typically been attributed to PCSS driven by conflict or male adaptations to fertilization and competition. Although PCSS plays a role in the evolution of the ejaculate (Birkhead 1995; Birkhead and Pizzari 2002; Perry et al. 2013; Wigby et al. 2020), our results show a large proportion of SFPs evolving under relaxed selection, as well as selective constraints. Conversely, we have found a relatively low proportion of SFPs evolving under positive selection, even after removing genes from the analysis to address the possibility of failing to detect positive selection among the shortest genes. Genes under positive selection were not limited to functions related to PCSS. These observations should caution about generalizations derived from results that focus on specific SFPs.

The differences observed in evolutionary rate of SFPs have often been linked to varying degrees of tissue specificity and groups of genes expressed in particular tissues (Dean et al. 2008, 2009; Finseth et al. 2014). We did find that SFPs that are not AG‐ or testes‐specific evolve primarily under selective constraints. Our findings are not an exception, as there have been reports of SFPs being conserved among species of mice (Dean et al. 2009), primates (Good et al. 2013), and birds (Finseth et al. 2014). Further, constrained SFP genes were overrepresented among older genes. It is tempting to speculate that these SFPs with nonreproductive tissue‐specific expression, and particularly those already present in the ancestor to the genus *Drosophila*, might be essential for housekeeping maintenance of reproduction.

Positive selection has traditionally been inferred through interspecific studies reporting *K*_a_/*K*_s_ ratios. However, such high ratios can also be predicted under relaxed selection (Dapper and Wade 2020). Interestingly, several genes previously reported as positively selected based on divergence data (Findlay et al. 2008) showed evidence for relaxed selection in our analyses. For example, out of 16 genes tested and identified as positively selected by Findlay et al. (2008), only three were confirmed based on our joint analysis of polymorphism and divergence, highlighting the importance of incorporating statistics that integrate polymorphism and divergence data to formally test selection at the molecular level (Kreitman 2000; Nielsen 2005). Nevertheless, our results support previous evidence of positive selection based on studies that used different populations as source material (Tsaur and Wu 1997; Aguadé 1998, 1999; Begun et al. 2000; Holloway and Begun 2004; Findlay et al. 2008; Wong et al. 2012). We confirmed only eight genes (*Acp26Aa*, *Acp29AB*, *antr*, *Qsox3*, *Sfp24Ba*, *Spn28F*, *Lectin30A*, and *CG31872*) as positively selected in RAL or ZI (only *Spn28F* in both), an intriguingly low number given the known functions in postmating fertilization success and sperm competitiveness. Among those, we find *Acp26Aa* (Ovulin), which stimulates ovulation and increases egg‐laying rate (Herndon and Wolfner 1995; Heifetz et al. 2000). A lectin gene, *Acp29AB*, is required for sperm storage and polymorphisms at this gene have been shown in association with a male's sperm competitive ability (Clark et al. 1995; Fiumera et al. 2005). The gene *Spn28F* encodes a protease inhibitor shown to be toxic to females when ectopically expressed (Mueller et al. 2007). Lastly, *CG31872* is an acid lipase encoding gene, which might have a role in providing energy for sperm motility (Walker et al. 2006), and has been found relevant for sperm offense ability (Reinhart et al. 2015).

Notably, postmating effect was not a category associated with positively selected SFP genes. For example, out of the 10 SFP genes for which there is unambiguous evidence of their role in sperm competition in *D. melanogaster* (Civetta and Ranz 2019), only two (*Acp26Aa* in ZI and *Acp29AB* in RAL) were found to be evolving under positive selection. One possible explanation for this lack of association might be a preponderance of nonadditive variation affecting the outcome of sperm competition in *Drosophila* and other species (Hughes 1997; Civetta and Ranz 2019). Moreover, if phenotypic responses mediated by SFPs are polygenic, individual SFPs might act as genes with minor effects and the molecular signals of selection only be detected by the combined action of multiple genes. Another possible contributing factor to this lack of association is that for other genes evolving under positive selection there is still absence of functional and phenotypic tests that could demonstrate their involvement in postmating mechanisms such as sperm competition (Civetta and Ranz 2019).

We found an excess of positively selected SFPs on the X‐chromosome. The hemizygous state of the X chromosome in males may allow for a faster accumulation, driven by positive selection, of recessive beneficial mutations (Charlesworth et al. 1987; Vicoso and Charlesworth 2006). There is a consistent pattern across a wide taxa spectrum for faster evolutionary divergence of sex chromosomes (X or Z) (i.e., faster‐X evolution) (Meisel and Connallon 2013; Garrigan et al. 2014; Kousathanas et al. 2014; Sackton et al. 2014; Jaquiéry et al. 2018) and the role of sex chromosomes in speciation (Coyne and Orr 1989, 2004; Good et al. 2008; Presgraves 2008). Further, we also found an excess of proteases in our positive selection class. Previous studies have documented rapid evolution for proteases that are components of seminal fluid in *Drosophila* (Kelleher et al. 2009), as well as in other insects (Andrés et al. 2006; Wong et al. 2008, 2012), birds (Rowe et al. 2020), and mammals (Good et al. 2013). Proteases are common in both male and female reproductive systems, being involved in the processing of other proteins known to be important in proper sperm storage and the stimulation of ovulation and egg‐laying (Kelleher et al. 2007; Takemori and Yamamoto 2009; LaFlamme et al. 2012). In some species, proteases are needed for proper acquisition of sperm motility (Friedländer et al. 2001; Zhao et al. 2012). Together, SFPs having proteolytic functions and/or located on X chromosome are promising candidates for further functional assays and speciation studies.

We found a preponderance of relaxed selected SFPs with an excess linked to male accessory glands‐specific expression. There are different reasons to expect relaxed selection to be predominant during the evolution of SFPs. First, selection intensity is potentially diminished because male‐specific genes are not under selection in females, that is, about half of the population (Pröschel et al. 2006; Dapper and Wade 2020), and, second, the predominant tissue‐biased expression of SFPs is consistent with reduced pleiotropy (Mank et al. 2008). In *Drosophila* and mice, the rapid evolution of sex‐biased genes is better explained by their narrow expression, with those limited to reproductive tissue evolving faster (Meisel 2011). A study in *Anastrepha* flies has shown evidence for a greater proportion of male‐biased, and reproductive‐biased, genes having signals of relaxed selection than unbiased genes (Congrains et al. 2018). Third, conditionally expressed genes often experience relaxed selection because of spatial and temporal fluctuations in the intensity of selection (Kawecki et al. 1997; Van Dyken and Wade 2010). SFPs might be particularly sensitive to social environment conditions. For example, it has been shown that *D. melanogaster* males adjusted the amount of two out of three SFPs tested in response to perceived male competition (Fedorka et al. 2011). Similarly, a larger survey of 58 SFPs in worms revealed a significant effect of mating group size on the relative expression of different transcripts (Patlar et al. 2019). A combination of effects such as reduced pleiotropy associated with sex‐ and tissue‐limited and condition‐dependent biases in expression might substantially contribute to the relaxation of selective pressures on SFPs.

Gene duplication is also very likely to have played an important role during the evolution of SFPs, contributing to their high divergence between species (Sirot 2019). In fact, some gene duplicates might experience periods of relaxed selection (Lynch and Conery 2000; Cardoso‐Moreira et al. 2016). Among the genes analyzed, we find a handful of multigene families with contrasting patterns in the mode of evolution of their paralogs. *Lectin‐29Ca*, *lectin‐30A*, and *Acp29AB* are closely related paralogs (Holloway and Begun 2004). *Acp29AB* is under positive selection in the Raleigh population, whereas its paralogs *lectin‐29Ca* and *lectin‐30A* are under relaxed selection. In Zambia, *Acp29AB* and *lectin‐29Ca* are under relaxed selection, whereas *lectin‐30A* has evolved under positive selection. *Acp53Ea*, Acp53C14a, *Acp53C14b*, and *Acp53C14c* are all paralogs, with *Acp53C14c* being more distantly related (Holloway and Begun 2004). In both populations, *Acp53C14a* is evolutionary constrained, whereas the paralogs are under relaxed selection. For another four Acp encoding genes (*Acp76A*, *CG31872*, *lectin‐46Cb*, and *Spn28F*) whose duplicates retain male accessory gland expression (Mueller et al. 2005), we found one duplicate evolutionary constrained with the others evolving under relaxed selection in both populations.

The comparisons of results between populations show similar patterns in terms of genes under common modes of selection despite different demographic histories. The number of positively selected and constrained genes in the ancestral (ZI) population that become relaxed in the derived population is in agreement with expectations of reduced efficacy of selection in derived populations (RAL) undergoing reduction in effective population size (Parsch et al. 2009). Interestingly, six genes under relaxed selection in ZI were found to be either constrained or under positive selection in RAL suggesting a possible role of local adaptation to new environment‐selective pressures. Further, the results obtained using noncategorical genomic features as predictors of mode of SFP evolution indicate that the classifier was not able to predict the gene category evolving under positive selection accurately in either population, which is to some extent expected due to the small number of observations available for that class. Lastly, although four of the five noncategorical genomic features showed significant differences among selection classes, their relative contribution was not consistent between populations, suggesting either no preeminent role for any of the genomic variables in predicting the mode of SFP evolution or population differences that call for further investigation.

Overall, our work contributes toward a better understanding of the causes of SFP gene evolution, proves the need of a more comprehensive sampling of SFPs before generalization about specific selective forces acting on them, and highlights the need to establish neutrality as the null hypothesis for formally testing the role of selection during the evolution of SFPs. Our analysis of patterns of polymorphism and divergence between the closely related species pair *D. melanogaster* and *D. simulans* allows us to draw conclusions about early stages of species divergence. However, it is important to acknowledge that patterns of evolution can be lineage specific and timescale dependent. For example, we identified genes *antr* and *CG9997* as positively selected and relaxed, respectively. These two genes are members of the Sex Peptide (SP) network. A study of the molecular evolution of SP network genes among species of the melanogaster group did not find evidence of positive selection for *antr*, but did for *CG9997* (McGeary and Findlay 2020). Moreover, *antr* was found to evolve under positive selection between *D. mojavensis* and *D. arizonae* (Bono et al. 2015). Similarly, in mammals, episodes of positive selection in phylogenetic studies can be recurrent or localized to specific clades or branches within a phylogeny (Finn and Civetta 2010; Grayson and Civetta 2013).

The identification of positively selected SFPs in a population‐specific context not only emphasizes the necessity of tests that incorporate polymorphism data, but also singles out putative targets for future functional assays. These assays could be directed to test the effect on fitness (e.g., fertility) and reproductive isolation by editing positively selected genes in *D. melanogaster* to mimic variants in its close relative *D. simulans*. Lastly, selection targets complex polygenic phenotypes, whereas population genetics tests gene's mode of evolution. Thus, a caveat, and a clear distinction to be made, is that the preponderance of nonadaptive molecular evolution of SFPs does not necessarily imply nonadaptive evolution of the traits they impact.

## AUTHOR CONTRIBUTIONS

BP collected data, conducted population and evolutionary genetics test statistics, and participated in discussions of the study design and writing of this article. VJ wrote code for a multiclass classifier to predict mode of evolution and provided comments on the writing. JR contributed to the analysis of data and participated in discussions of the study design, data gathering, and writing. AC contributed to the analysis of data and data gathering and participated in discussion of the study design and writing of this article.

## DATA ARCHIVING

Data and supplementary code openly available in Dryad at https://doi.org/10.5061/dryad.rjdfn2zbg.

## CONFLICT OF INTEREST

The authors declare no conflict of interest.

Associate Editor: Dr. Nadia Singh

Handling Editor: Dr. Andrew McAdam

## Supporting information

**Table S1**. Complete list of SFPs grouped by selection class classification for Zambia and Raleigh populations.
**Table S2**. Effect of eliminating an increasing proportion of the shortest genes on the fraction of genes evolving under relaxed selection in relation to the total considering both relaxed and positive selection.
**Table S3**. Standardized residuals of the *χ*^2^ tests performed to test for independence between the phylogenetic gene age and the mode of evolution of SFP encoding genes.
**Table S4**. Patterns of nonrandom association for expression specificity trends and different gene categories based on their mode of molecular evolution.
**Table S5**. Noncategorical feature differences among the three selection classes.
**Figure S1**. Sequence divergence (*D_xy_* and *K*_a_/*K*_s_) for SFPs (blue) and the rest of the genome (yellow) for Zambia and Raleigh populations.
**Figure S2**. Observed levels of polymorphism *π*_a_/*π*_s_ and divergence *K*_a_/*K*_s_ for SFP coding genes in Zambia (A) and Raleigh (B) populations.
**Figure S3**. Phylogenetic origin of SFP encoding genes.
**Figure S4**. Distribution of accuracy values in predicting the selection class of SFP genes in Zambia and Raleigh populations.

**Table S1**. List of SFPs grouped by selection class classification for Zambia and Raleigh populations.
