# Supplementary material for: Nonadaptive molecular evolution of seminal fluid proteins in Drosophila
Source: Evolution. 2021 Jul 9;75(8):2102–13. doi: 10.1111/evo.14297 (PMC8457112; doi:10.1111/evo.14297)

**SUPPLEMENTARY TABLES AND FIGURES**

**Table S1:** **Complete list of SFPs grouped by selection class classification for Zambia and Raleigh populations**. The list includes SFPs for which ratios (K_a_/K_s_) and eMKT could not be determined due to K_s_= 0 or lack of polymorphism, respectively. For categorical values 0 is used for “No” and 1 for “Yes”.

| **Table S2. Effect of eliminating an increasing proportion of the shortest genes on the fraction of genes evolving under relaxed selection in relation to the total considering both relaxed and positive selection** | | | |
| --- | --- | --- | --- |
| **Fraction of Genes Removed *** | **Genes Relaxed Selection / Total †** | ***P* (Difference In Proportions) ¦** | ***P* (Difference In Length) ‡** |
| *ZI* |  |  |  |
| 0% | 124 / 155 = 0.8000 | na | 0.0001 |
| 5% | 117 / 148 = 0.7905 | 0.9509 | 0.0004 |
| 10% | 110 / 141 = 0.7801 | 0.7823 | 0.0013 |
| 20% | 98 / 129 = 0.7597 | 0.5000 | 0.0114 |
| 33% | 75 / 103 = 0.7282 | 0.2324 | 0.0512 |
| 50% | 53 / 77 = 0.6883 | 0.0855 | 0.3671 |
| *RAL* |  |  |  |
| 0% | 145 /162 = 0.895 | na | 0.0097 |
| 5% | 138 / 155 = 0.890 | 1 | 0.0179 |
| 10% | 131 / 148 = 0.885 | 0.9224 | 0.0333 |
| 20% | 117 / 134 = 0.873 | 0.6849 | 0.1154 |
| 33% | 92 / 107 = 0.860 | 0.4955 | 0.1643 |
| 50% | 68 / 80 = 0.850 | 0.4208 | 0.2227 |

***** Out of 254 genes regardless of their mode of molecular evolution.

**†** Total, sum of the number of genes evolving under relaxed selection and positive selection.

**¦** According to two sample tests for equality of proportions with continuity correction. The test uses as a reference the proportion of genes evolving under relaxed selection prior to removing any gene.

**‡** Between the genes evolving under relaxed selection and positive selection according to the Wilcoxon signed-rank test.

| **Table S3. Standardized residuals of the χ^2^ tests performed to test for independence between the phylogenetic gene age and the mode of evolution of SFP encoding genes** | | | | | |
| --- | --- | --- | --- | --- | --- |
|  | **Phylogenetic Age Class *** | | | | |
| **Evolution Mode** | **Recent** | ***mel* subgroup** | ***mel* group** | ***Sophophora*** | **Ancient** |
| ***Zambia*** |  |  |  |  |  |
| **Positive** | -0.698 | -1.099 | 1.364 | 0.672 | -0.469 |
| **Relaxed** | **2.015** | **4.113** | 0.597 | 1.234 | **-4.633** |
| **Constrained** | -1.526 | **-3.342** | -1.545 | -1.7 | **4.953** |
| ***Raleigh*** |  |  |  |  |  |
| **Positive** | -0.475 | 0.859 | 0.113 | -0.748 | -0.039 |
| **Relaxed** | 1.703 | **2.71** | **2.137** | 1.488 | **-4.814** |
| **Constrained** | -1.481 | **-3.187** | **-2.223** | -1.124 | **4.897** |

Absolute values of standardized residuals > 1.967 denote a significant contribution to the χ^2^ obtained at a nominal *P* value < 0.05. Positive values, overrepresentation; negative values; depletion.

* Age classes are color coded according to Fig. S3. The class *Recent* refers to genes whose phylogenetic origin was inferred to happen in the ancestor to *D. simulans, D. sechellia,* and *D. melanogaster* or just in the latter species after it branched off from the ancestor to the first two species. The class *Ancient* refers to genes whose phylogenetic origin was inferred to be in the ancestor to the two main subgenera in the genus *Drosophila.*

**Table S4: Patterns of non-random association for expression specificity trends and different gene categories based on their mode of molecular evolution**

|  | | **Selection Regime** | | | | | |
| --- | --- | --- | --- | --- | --- | --- | --- |
|  |  | **Constrained** | | **Positive** | | **Relaxed** | |
| **Contrast** | ***P*-adj *** | **Odds Ratio** | ***P*-adj †** | **Odds Ratio** | ***P*-adj †** | **Odds Ratio** | ***P*-adj †** |
| ***Zambia*** |  |  |  |  |  |  |  |
| **Accessory gland-specific vs testes-specific** | 0.074 | 0.387 | 0.185 | 0.477 | 0.386 | 3.846 | 0.074 |
| **Accessory gland-specific vs others** | **<0.001** | 0.230**↓** | **<0.001** | 1.090 | 1.000 | 4.231**↑** | **<0.001** |
| **Testes-specific vs others** | 0.439 | 0.596 | 0.575 | 2.284 | 0.575 | 1.100 | 1.000 |
|  |  |  |  |  |  |  |  |
| ***Raleigh*** |  |  |  |  |  |  |  |
| **Accessory gland-specific vs testes-specific** | 0.727 | 0.675 | 0.904 | inf | 0.904 | 1.053 | 1.000 |
| **Accessory gland-specific vs others** | **<0.001** | 0.288**↓** | **<0.001** | 2.310 | 0.160 | 2.582**↑** | **0.002** |
| **Testes-specific vs others** | 0.355 | 0.413 | 0.234 | 0.000 | 1.000 | 3.021 | 0.234 |

Others includes other-tissue specific and non-tissue specific SFPs.

Only the 254 SFPs common to the populations of Zambia and Raleigh are considered.

* For each contrast, genes are split into two categories and differential association is tested with modes of evolution by using a 2x3 Fisher exact test (FET). When significant, the *P-*value is bolded.

† *Posthoc* 2x2 FETs to test for significant excess (odds ratio>1) or deficit (odds ratio<1) between any selective regime and the other two. *P-*values are FDR corrected. When significant, the *P-*value is bolded, and an arrow identifies the excess or deficit for the first category listed. For the alternative category the pattern is the opposite.

**Table S5: Non-categorical feature differences among the three selection classes**. *P*-values (bolded when significant) were obtained for each feature from one-way analysis of variance tests.

|  | **Population** | |
| --- | --- | --- |
| **Genomic feature** | **Zambia** | **Raleigh** |
|  | ***P*-values** | ***P*-values** |
| Gene length | **0.016** | **0.015** |
| Number of transcripts | **<0.001** | **<0.001** |
| Effective number of codons | 0.370 | 0.276 |
| Tau Index | **<0.001** | **<0.001** |
| Recombination frequency | **0.032** | **0.031** |

**Figure S1:** **Sequence divergence (D_xy_ and K_a_/K_s_) for SFPs (blue) and the rest of the genome (yellow) for Zambia and Raleigh populations**. The box plots show the distribution of the accuracy values. Boxes represent the interquartile range (IQR) around the median (horizontal black line). The whiskers extend to 1.5 times the IQR and incorporate the accuracy values as solid dots. Outlier data points are shown below and above the whiskers. Red dots are mean values.


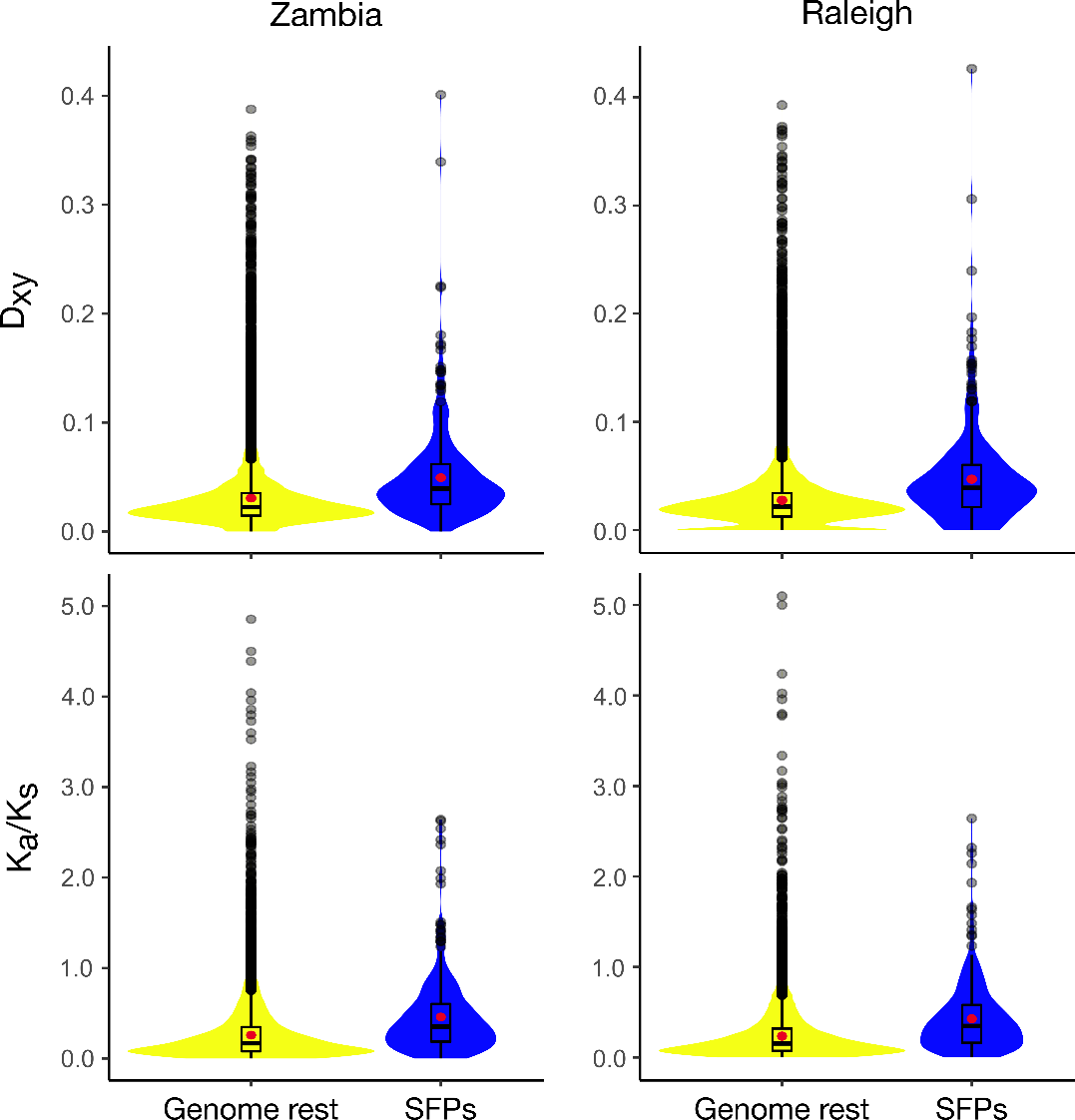


**Figure S2:** Observed levels of polymorphism πa/πs and divergence K_a_/K_s_ for SFP coding genes in Zambia (A) and Raleigh (B) populations. The pink dashed line represents neutrality, where polymorphism equals divergence. The blue dashed line is the observed relationship between polymorphism and divergence.


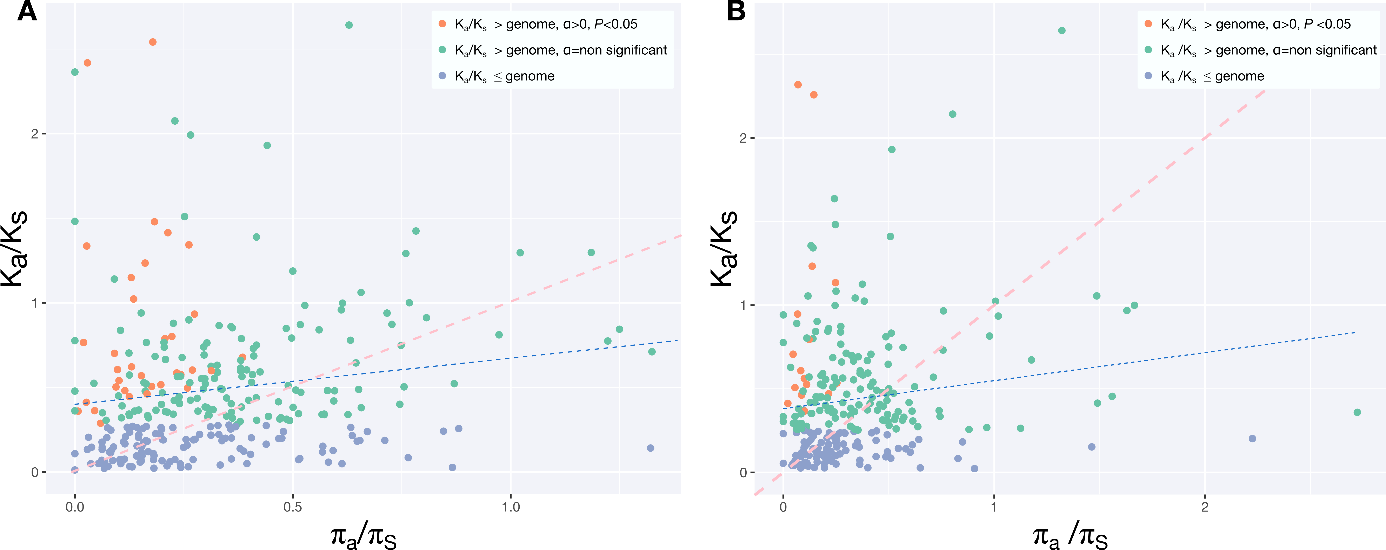


**Fig. S3. Phylogenetic origin of SFP encoding genes.** The branch of origin of each gene was as reported (Xia et al.). Five age classes, which are color coded, were established based on genes present in the ancestor of: the genus *Drosophila* (branch 0); the subgenus *Sophophora* (branches 1 and 2); the *melanogaster* group (branch 3); the *melanogaster* subgroup (branch 4); and the simulans complex and *D. melanogaster,* or only in the latter (branches 5 and 6). Only 258 SFP genes for which the branch assignment was reliable performed in Xia et al. are shown. In parenthesis, the observed *vs*. expected counts for each age class are indicated. The expected counts are proportional to the representation of each class in the whole set of genes for which their phylogenetic origin was reliably inferred, *i.e.* 13,083 (Xia et al.). The asterisk indicates the branch for which its corresponding standardized residual in the χ^2^ performed was > 1.967, *i.e.* when the age class has a significant contribution to the difference between expected and observed gene counts.


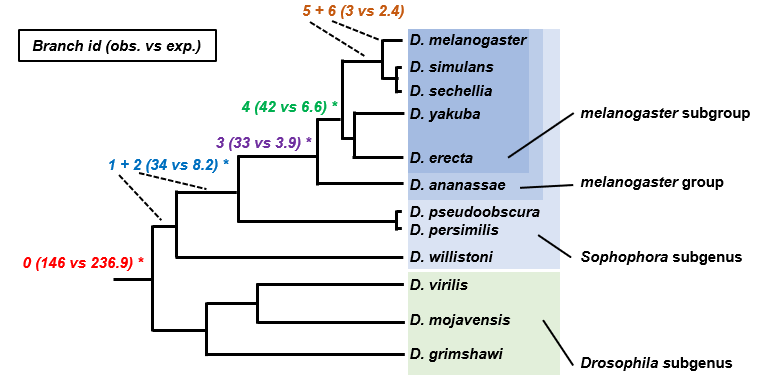


**Figure S4:** **Distribution of accuracy values in predicting the selection class of SFP genes in Zambia and Raleigh populations**. Prediction accuracy of the most optimal multinomial regression model given the predictors ultimately considered (number of transcripts, Tau index, recombination rate, and gene length) over 100 cross-validation experiments. The results are shown when all three modes of evolution are considered jointly and when examined separately. As an evaluation metric, we used the ratio M/N, where N denotes the number of observations in the test set and M denotes the number of observations whose selection class was predicted correctly.


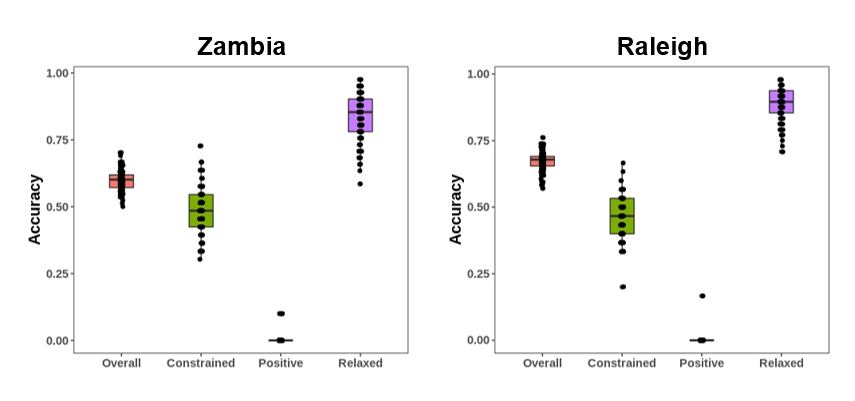

Supplement: Supplementary file 1 — Table S1. Complete list of SFPs grouped by selection class classification for Zambia and Raleigh populations. Table S2. Effect of eliminating an increasing proportion of the shortest genes on the fraction of genes evolving under relaxed selection in relation to the total considering both relaxed and positive selection. Table S3. Standardized residuals of the χ2 tests performed to test for independence between the phylogenetic gene age and the mode of evolution of SFP encoding genes. Table S4. Patterns of nonrandom association for expression specificity trends and different gene categories based on their mode of molecular evolution. Table S5. Noncategorical feature differences among the three selection classes. Figure S1. Sequence divergence (Dxy and K a/K s) for SFPs (blue) and the rest of the genome (yellow) for Zambia and Raleigh populations. Figure S2. Observed levels of polymorphism πa/πs and divergence K a/K s for SFP coding genes in Zambia (A) and Raleigh (B) populations. Figure S3. Phylogenetic origin of SFP encoding genes. Figure S4. Distribution of accuracy values in predicting the selection class of SFP genes in Zambia and Raleigh populations. [file EVO-75-2102-s002.docx]
